# Trastuzumab and fulvestrant combination therapy for women with advanced breast cancer positive for hormone receptor and human epidermal growth factor receptor 2: a retrospective single-center study

**DOI:** 10.1186/s12885-021-09128-1

**Published:** 2022-01-04

**Authors:** Yukinori Ozaki, Yosuke Aoyama, Jun Masuda, Lina Inagaki, Saori Kawai, Tomoko Shibayama, Tetsuyo Maeda, Mami Kurata, Kazuyo Yoshida, Sumito Saeki, Mari Hosonaga, Ippei Fukada, Fumikata Hara, Takayuki Kobayashi, Kokoro Kobayashi, Satoshi Miyake, Toshimi Takano, Takayuki Ueno, Shinji Ohno

**Affiliations:** 1 Breast Oncology Center, The Cancer Institute Hospital of Japanese Foundation for Cancer Research, 3-8-31 Ariake, Koto-ku, Tokyo, 135-8550 Japan; 2 Department of Clinical Oncology, Graduate School of Medical and Dental Sciences, Tokyo Medical and Dental University, Tokyo, Japan; 3 Department of Medical Oncology, Toranomon Hospital, Tokyo, Japan

**Keywords:** Trastuzumab, Fulvestrant, Hormone receptor-positive HER2-positive breast cancer

## Abstract

**Background:**

Trastuzumab and fulvestrant combination therapy is one of the treatment options for patients with hormone receptor- and human epidermal growth factor receptor 2 (HER2)-positive metastatic breast cancer; however, there are limited studies evaluating the efficacy of this combination therapy.

**Methods:**

We retrospectively reviewed the data of women with hormone receptor- and HER2-positive metastatic breast cancer who received trastuzumab and fulvestrant combination therapy between August 1997 and August 2020 at the Cancer Institute Hospital. The primary endpoint of this study was progression-free survival, and the secondary endpoints were response rate, overall survival and safety.

**Results:**

We reviewed the data of 1612 patients with recurrent or metastatic breast cancer, of which 118 patients were diagnosed with hormone receptor- and HER2-positive breast cancer. Of these, 28 patients who received trastuzumab and fulvestrant combination therapy were eligible for this study. The median treatment line for advanced breast cancer was 6 (range, 1–14), the median progression-free survival was 6.4 months (95% confidence interval [CI], 3.46–8.17), and the median overall survival was 35.3 months (95% CI, 20.0–46.7). Of the 28 patients, partial response was observed in 1 (4%), stable disease in 17 (61%), and progressive disease in 10 (36%) patients. The disease control rate was 64%. Adverse events of grade ≥ 3 were not observed.

**Conclusions:**

Trastuzumab and fulvestrant combination therapy showed moderate clinical efficacy and no severe toxicity after standard anti-HER2 treatment, which is a reasonable treatment option for patients with hormone receptor- and HER2-positive metastatic breast cancer. These data contribute to understanding the efficacy of trastuzumab and fulvestrant combination therapy as control data for further development of anti-HER2 agents plus hormone therapy.

## Background

Breast cancer is the most common cancer among women and the second most frequent newly diagnosed cancer worldwide [1]. Breast cancer has been divided into subtypes depending on the presence of hormone receptors (HRs) for estrogen and progesterone and HER2 expression [2]. Accordingly, there are four major subtypes of breast cancer: HR-positive HER2-negative (HR + HER2−), HR-positive HER2-positive (HR + HER2+), HR-negative HER2-positive (HR − HER2+), and HR-negative HER2-negative (triple-negative breast cancer). The HER2-positive subtype accounts for 15–20% of all breast cancer subtypes [3]. Approximately half of the HER2-positive breast cancers express HRs [4]. HR + HER2+ breast cancer account for approximately 10% of all breast cancer cases [5, 6].

The standard first-line systemic treatment for metastatic HER2-positive breast cancer is chemotherapy with pertuzumab plus trastuzumab plus taxane [7]. Endocrine plus HER2-targeted therapy is a treatment option for patients with HR + HER2+ metastatic breast cancer. Previous clinical trials have showed that a combination of aromatase inhibitor and HER2-targeted therapy has greater clinical benefits than endocrine therapy alone in patients with HR + HER2+ breast cancer [8–10]. In the case of disease progression under aromatase inhibitor and HER2-targeted combination therapy, fulvestrant with HER2-targeted therapy is a reasonable treatment option; however, to our knowledge, the efficacy of fulvestrant and trastuzumab combination therapy has not been reported. Here, we retrospectively reviewed the efficacy and safety of fulvestrant and trastuzumab combination therapy at our hospital.

## Methods

This study was designed as a retrospective review of the medical records of patients with advanced breast cancer who received systemic therapy between January 2001 and September 2020 at our institution. Patients with HR + HER2+ metastatic breast cancer were selected and those treated with fulvestrant and trastuzumab combination therapy were identified. HR and HER2 were assessed using tumor immunohistochemistry (IHC) and/or fluorescence in situ hybridization. Clinicopathological data, including age, HR and HER2 status, clinical and pathological stages, prior treatment of recurrent or metastatic breast cancer, metastatic organ site, response, treatment outcome, and survival, were collected from the medical records. Tumors were defined as HR+ when IHC showed an Allred score ≥ 3 for estrogen and/or progesterone receptor, according to American Society of Clinical Oncology/College of American Pathologists Guideline [11]. Tumor HER2 status was defined as positive when IHC for HER2 showed a score of 3+ or 2+ with gene amplification confirmation by fluorescence in situ hybridization. Patients received trastuzumab intravenously at 8 mg/kg on day 1 of cycle 1, and this was maintained at 6 mg/kg on day 1 of all subsequent 21-day cycles. Fulvestrant was intramuscularly administered at 500 mg on days 1 and 15 of cycle 1 and then once every 4 weeks.

### Endpoints

The primary endpoint of this study was progression-free survival (PFS), and the secondary endpoints were overall survival (OS) and safety. In the analysis of survival data, 95% CIs were estimated using the exact method. To evaluate the association between survival data and clinicopathological features, the chi-square test was used. A *P* value of < 0.05 was considered statistically significant. Statistical analysis was performed using JMP version 14.3.0 (SAS Institute Inc., Cary, NC, USA) and SPSS Statistics 27.0 (IBM corporation, New York, USA). This study was approved by the institutional review board (# 2020–1280). Comprehensive consent was obtained from accusable patients in our institution.

## Results

The data of 1612 patients with recurrent or metastatic breast cancer during the targeted period were reviewed and 118 patients with histologically documented HR + HER2+ breast cancer were identified. Of these, 28 patients received trastuzumab and fulvestrant combination therapy.

The patient characteristics are presented in Table 1. The median age was 66 (range, 50–80) years. Of these 28 patients, 7 (25%) had de novo stage IV and 21 (75%) had recurrent disease. Before trastuzumab and fulvestrant combination therapy, the median treatment line for advanced breast cancer was 6. Six patients had a treatment line of ≤2, and the remaining 22 patients were treated on the third treatment line or later. Trastuzumab was previously administered for advanced breast cancer to all patients, pertuzumab to four patients, and trastuzumab emtansine (T-DM1) to 10 patients. Of all patients, 21 (75%) had visceral metastasis, including liver and brain metastases.

**Table 1:** Patient characteristics

Characteristics	***N*** = 28 (%)
Age (years)	
median	66 (range: 50–80)
Stage	
De novo stage IV	7 (25)
recurrent disease	21 (75)
Subtype	
HR+ HER2+	28 (100)
others	0
Number of treatment line	
median	6
1 or 2	6 (21)
3–6	9 (32)
7–10	9 (32)
> 10	4 (14)
Prior treatment for recurrent or metastatic breast cancer	
Trastuzumab	28 (100)
Pertuzumab	4 (14)
T-DM1	10 (36)
Trastuzumab deruxtecan (T-DXd)	2 (7)
Visceral metastasis	
Yes	21 (75)
No	7 (25)
Liver metastasis	
Yes	11 (39)
No	17 (61)
Brain metastasis	
Yes	4 (14)
No	24 (86)

### Efficacy

**Fig. 1:**
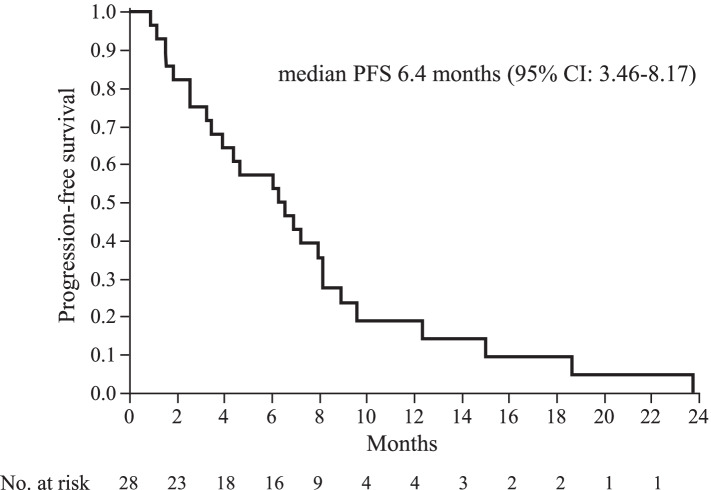
Progression-free survival in all patients. Kaplan–Meier curve of progression-free survival (PFS) of fulvestrant and trastuzumab combination therapy in patients with hormone receptor- and human epidermal growth factor receptor 2-positive metastatic breast cancer

**Fig. 2:**
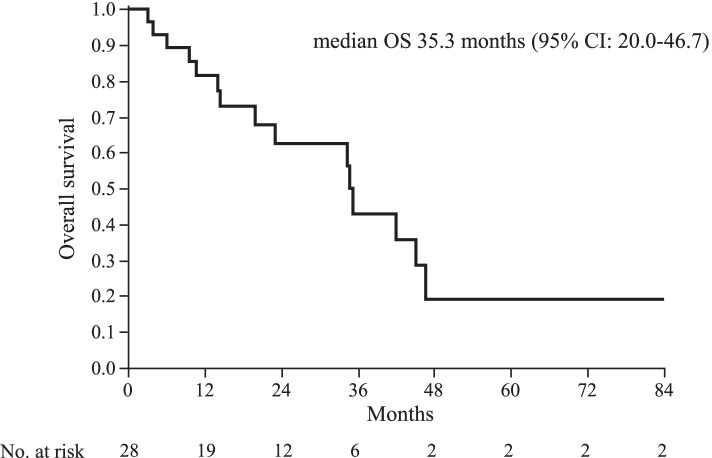
Overall survival in all patients. Kaplan–Meier curve of OS of fulvestrant and trastuzumab combination therapy in patients with hormone receptor- and human epidermal growth factor receptor 2-positive metastatic breast cancer

**Fig. 3:**
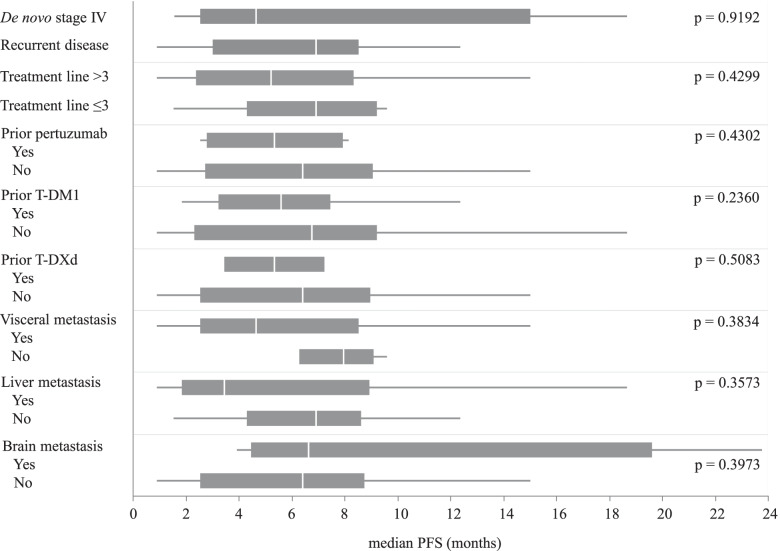
Subgroup analysis of PFS by patient characteristics. Forest plots of PFS evaluated in subgroups depending on the clinicopathological features

**Fig. 4:**
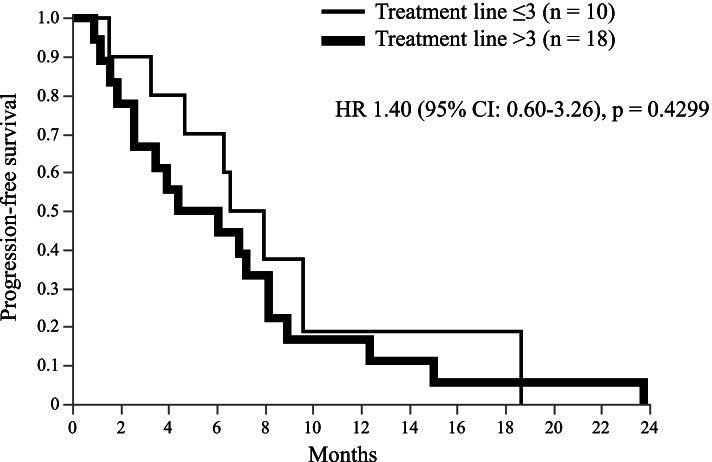
PFS subgroup analysis by number of treatment line. Kaplan–Meier curves of PFS in patients who received fulvestrant and trastuzumab combination therapy as ≤3 line treatment or > 3 line treatment

**Fig. 5:**
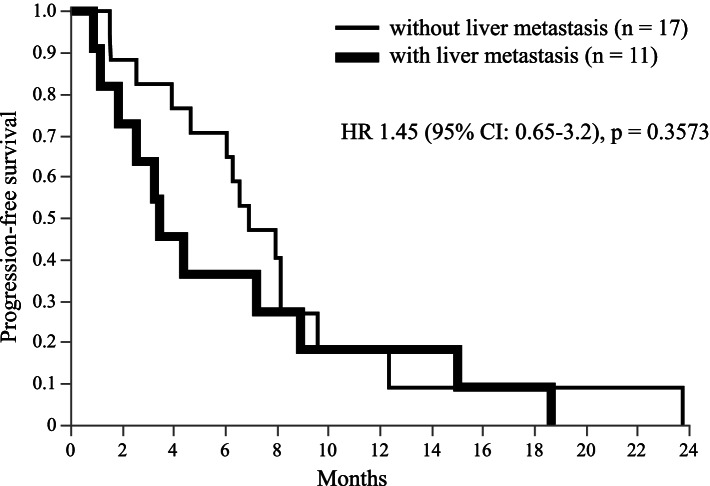
PFS subgroup analysis in patients with or without liver metastasis. Kaplan–Meier curves of PFS in patients who had liver metastasis or not

Complete response (CR) was not observed in any patient. Partial response (PR) was observed in 1, stable disease (SD) in 17, and progressive disease in 10 patients. The overall response rate was 4%, and the disease control rate (DCR = CR + PR + SD) was 64%. The median PFS was 6.4 months (95% CI, 3.46–8.17), and median OS was 35.3 months (95% CI, 20.0–46.7) (Figs. 1 and 2). Subgroup analysis depending on patient characteristics is shown in Fig. 3, which suggested that no significant difference in PFS was observed in any subgroup analysis. PFS in patients who received trastuzumab and fulvestrant combination therapy as ≤3rd line treatment or did not have liver metastasis tended to be better compared to those in patients who received the treatment in later line or with liver metastasis. Median PFS was 7.3 months (95% CI, 1.53–18.67) and median OS was not achieved in patients receiving trastuzumab and fulvestrant combination therapy as ≤3rd line treatment. In contrast, the median PFS was 5.2 months (95% CI, 2.57–8.17), and median OS was 34.8 months (95% CI, 14.5–45.1) in patients who received the therapy as >3rd line treatment. The PFS and OS of this subgroup were not significantly different (log-rank test, *p* = 0.4299 and *p* = 0.2550, respectively) (PFS data in Fig. 4). Additionally, PFS in patients with liver metastasis tended to be worse than that in patients without liver metastasis, and median PFS was 3.5 months (95% CI, 1.17–8.93) and 6.9 months (95% CI, 3.93–8.17), respectively (log rank test, *p* = 0.3573) (Fig. 5). No adverse events of ≥grade 3 were observed.

## Discussion

Trastuzumab and fulvestrant combination therapy showed a median PFS of 6.4 months, median OS of 35.3 months, and 64% DCR, which suggested moderate efficacy in patients with HR + HER2+ metastatic breast cancer. In this study, most patients were heavily pretreated and developed visceral metastasis, including liver and brain metastases, indicating poor prognosis in these patients. The response rate was only 4%, which might be due to the late treatment line of this population; the median number of treatment lines was six, and four patients were treated after the 10th line.

Real-world evidence of treatment after T-DM1 in patients with HER2+ metastatic breast cancer showed that most patients received trastuzumab and chemotherapy after T-DM1, and approximately 5% of the patients received hormone therapy with or without anti-HER2 antibodies [12]. The median PFS after T-DM1 treatment was 6.1 months (95% CI, 5.3–6.7) and median OS was 23.7 (95% CI, 20.7–27.4), which are comparable with the PFS and OS of trastuzumab and fulvestrant combination therapy in the current study. This suggests that trastuzumab and fulvestrant combination therapy is a reasonable treatment option after T-DM1 for patients with HR + HER2+ metastatic breast cancer.

Recently, clinical trials on the addition of a CDK4/6 inhibitor to the combination of fulvestrant and HER2-targeted therapy have been conducted in patients with HR + HER2+ breast cancer [13, 14]. However, the data on fulvestrant and trastuzumab combination therapy that should have been the background for these studies are scarce; hence, our data are considered useful. The monarcHER trial, a phase 2 trial that compared abemaciclib plus trastuzumab plus fulvestrant, abemaciclib plus trastuzumab, and standard-of-care chemotherapy plus trastuzumab, showed better PFS with abemaciclib plus trastuzumab plus fulvestrant than with standard-of-care chemotherapy plus trastuzumab [13]. The median PFS of the abemaciclib plus trastuzumab plus fulvestrant group (*n* = 79) and that of standard-of-care chemotherapy and trastuzumab group were 8.3 months (95% CI, 5.9–12.6) and 5.7 (95% CI, 5.4–7.0), respectively. The PFS of trastuzumab plus fulvestrant in our study was comparable to that of standard-of-care chemotherapy and trastuzumab in the monarcHER trial. The patient background in our study was also similar to that in the monarcHER trial. For example, patients who received at least two HER2-targeted therapies for advanced breast cancer were included. In the abemaciclib plus trastuzumab plus fulvestrant group, 44% patients had two or three previous systemic therapies for advanced breast cancer and 56% had four or more. Moreover, 73% patients had visceral metastasis, and most patients were heavily pretreated for it. The overall response rate in the abemaciclib plus trastuzumab plus fulvestrant group was 33%, which was higher than that in our survey. This suggests that adding abemaciclib to trastuzumab and fulvestrant combination therapy in this population is effective. Based on these efficacy data, anti-HER2 therapy plus endocrine plus CDK4/6 inhibitor combination is a promising strategy in this patient population and phase 3 trials are ongoing. For example, DETECT V/CHEVENDO trial is recruiting patients, which is a randomized phase III study comparing the safety and efficacy of trastuzumab plus pertuzumab and the ribociclib, CDK4/6 inhibitor, along with either endocrine therapy or chemotherapy and includes trastuzumab plus pertuzumab plus ribociclib plus fulvestrant arm (NCT02344472). Another phase III trial, PATINA, is evaluating the efficacy of adding palbociclib to the anti-HER2 therapy plus endocrine therapy after 4 to 8 cycles of induction treatment (NCT02947685).

There has recently been remarkable progress in the development of new drugs for HER2+ metastatic breast cancer [15]. These new drugs include novel antibody–drug conjugates (trastuzumab deruxtecan) and tyrosine kinase inhibitors (neratinib and tucatinib). Some of these agents are being developed in clinical trials as combination therapy with fulvestrant; however, there are no control efficacy data on trastuzumab and fulvestrant combination therapy. This study contributes to understanding the efficacy of trastuzumab and fulvestrant combination therapy and further development of anti-HER2 agents plus hormone therapy.

We recognized several imitations of this study. First, this is a single-center retrospective study, which makes it difficult to appropriately evaluate adverse events or toxicity. Second, only 14% patients previously received treatment containing pertuzumab. The records include patients since 2001, which implies that most patients received anti-HER2 treatment before pertuzumab approval. Finally, the number of patients who received trastuzumab and fulvestrant was small, which requires careful interpretation of the results.

## Conclusions

Trastuzumab and fulvestrant combination therapy showed moderate clinical efficacy, which is a reasonable option for HR + HER2+ metastatic breast cancer after standard anti-HER2 therapy. These data contribute to understanding the efficacy of trastuzumab and fulvestrant combination therapy as control data for further development of anti-HER2 agents plus hormone therapy.

## Data Availability

The datasets used and/or analyzed during the current study available from the corresponding author on reasonable request.

## Abbreviations

HER2: Human epidermal growth factor receptor 2
CI: Confidence interval
HR: Hormone receptor
IHC: Immunohistochemistry
PFS: Progression-free survival
OS: Overall survival
T-DM1: Trastuzumab emtansine
CR: Complete response
PR: Partial response
SD: Stable disease
